# *In vitro* and *in vivo* anticancer activity of Lycorine in prostate cancer by inhibiting NF-κB signaling pathway

**DOI:** 10.7150/jca.75597

**Published:** 2022-08-21

**Authors:** Jie Liu, Shengjun Sun, Cheng Zhou, Zhihong Sun, Qin Wang, Chengming Sun

**Affiliations:** 1 Yantai Yuhuangding Hospital, Yantai, P. R. China.; 2 Yantaishan Hospital of Yantai, P. R. China.; 3 School of Life Sciences, Lanzhou University, Lanzhou, P. R. China.; 4 Shenzhen Bay laboratory. Shenzhen, P. R. China.

**Keywords:** Lycorine, NF-κB, prostate cancer, apoptosis, tumor growth

## Abstract

NF-κB transcription factors critically regulate the expression of genes which are involved in important cellular processes, including cellular proliferation and apoptosis. Abnormal activation of the NF-κB signaling pathway has been implicated in a variety of human cancers. Hyper-activation of the NF-κB signaling pathway has been found to lead to tumor survival, anti-apoptosis and invasion in the development of prostate cancer. In the present work, we identified Lycorine as a potent NF-κB inhibitor using a NF-κB activity dependent luciferase reporter in PC3 and DU145 prostate cancer cells. With this reporter gene assay, we found that Lycorine significantly suppressed the constitutive NF-κB activity as well as the NF-κB activity induced by TNF-α, LPS, PMA and IL-1β. Western blotting analysis of the NF-κB signaling pathway further showed that Lycorine inhibited IκB-α (inhibitor of κB) phosphorylation, IκB-α degradation, and p65 phosphorylation. Consistent with this, the subsequent nuclear translocation of p65 was blocked by Lycorine as evidenced in the immunofluorescence assay and western blotting. Furthermore, we observed that cell cycle was arrested at G2/M in Lycorine treated cells using FACS analysis. Western blotting analysis indicated that Lycorine increased the expression of Cyclin D1 but decreased the expression of p21. In addition, FACS analysis showed that Lycorine induced apoptosis in DU145 and PC3 cells. Western blotting analysis revealed that Lycorine decreased the expression of anti-apoptosis genes myc, survivin and Bcl-2 while increased cleavage of PARP. Finally, we observed a significant anticancer effect of Lycorine in a RM-1 prostate cancer xenograft mouse model. In agreement with its *in vitro* anticancer effect, Lycorine inhibited p65 phosphorylation, IKK-β phosphorylation and the expression of Ki-67, while increased the cleavage of Caspase 3 in tumor tissue. Taken together, our data demonstrated the *in vitro* and *in vivo* anti-prostate cancer activity of Lycorine by inhibiting the NF-κB signaling pathway, and highlighted it as a lead compound for further development into an effective anticancer drug.

## Introduction

In addition to its role in the regulation of immune and inflammatory response, the nuclear transcription factor-kappa B (NF-κB) signal pathway has been found to be critically important for progression and development of cancer 1-4. In mammals, NF-κB family has five different members: Rel A (p65), Rel B, C-Rel, NF-κB1/p50, and NF-κB2/p52 5. NF-κB members bind to κB site as homodimers or heterodimers, which can suppress or activate transcription of target genes. In canonical NF-κB signaling pathway, p50/p65 heterodimer are retained in cytoplasm by interacting with inhibitor of κB (IκB) in unstimulated cells 6, 7. Up on stimulation like TNF-α, IκB undergoes phosphorylation by its upstream IκB kinase (IKK) complex and then ubiquitination, and degradation by 26S proteasome 8. Activated heterodimers p50/p65 is released and translocated into the nucleus where it induces transcription of many genes involved in cell growth, adhesion, survival and anti-apoptosis.

Aberrant and constitutive activation of NF-κB has been reported in many human cancers, such as prostate cancer, breast cancer, lymphoma, and glioma 10-12. Hyper NF-κB activity can lead to the progression of prostate cancer by regulating its target genes that promote cancer cell growth, proliferation and survival. Moreover, hyper NF-κB activity are frequently associated with angiogenesis, invasion and metastasis, which may further contribute to the progression of prostate cancer. Therefore, inhibiting NF-κB signaling pathway is a potential therapeutic strategy for treatment of prostate cancer.

Lycorine, a nature product extracted from Lycoris, has a range of biological activities, including anti-viral, anti-malarial, anti-tumor and anti-inflammation. Lycorine has been found to inhibit cell proliferation, arrest cell cycle and induce apoptosis in human cancer cells 13, 14,15. The previous study has figured out Lycorine's anti-proliferative and anti-migratory properties for prostate cancer in many prostate cancer cell lines, which may prevent EGF-induced JAK/STAT signaling via dependent on STAT expression 16. However, its molecule mechanism of inhibiting cancer remains unclear. In the present work, we determined its effect on NF-κB signaling using a prostate cancer PC3-NF-κB-Luc cell line that stably expresses NF-κB promoter driving luciferase reporter. With this cell lines, we identified Lycorine as a potent NF-κB signaling pathway inhibitor in prostate cancer. We demonstrated that Lycorine could suppress the constitutive NF-κB signaling as well as the induced NF-κB signaling, inhibit cell growth and migration, and induce cell cycle arrest and apoptosis in prostate cancer cells. Moreover, we observed that Lycorine inhibited the tumor growth in a RM-1 prostate cancer xenograft mouse model by inhibiting NF-κB signaling activity and inducing cell apoptosis. Finally, our data provided mechanistic insights into anticancer effect of Lycorine in prostate cancer and its potential involvement in the development of novel NF-κB inhibitor.

## Materials and Methods

### Reagents and Cell culture

Antibodies against P-p65, p65, P-IκB-α, IκB-α, Bcl-2, survivin, c-Myc, and PARP were purchased from Cell Signaling Technology (Beverly, MA, USA). GenEscort^TM^ Transfection Reagent was purchased from Wisegen Biotechnology Corporation (Nanjing, China). TNF-α was purchased from Pepro Tech (London, UK). Lycorine was obtained from Tianjin science and technology corporation (Tianjin, China). TPCA-1 (a specific inhibitor of NF-κB), PMA, LPS, and IL-1β were purchased from Sigma Aldrich (St. Louis, MO, USA). DU145 (human prostate cancer), PC3 (human prostate cancer) and HEK293T (human embryonic kidney) cells were obtained from the American Type Culture Collection. PC3 cells stably expressing NF-κB regulated luciferase reporter (PC3-NF-κB-Luc) was established in our Lab 16. Except for HEK293T cells, which were grown in Dulbecco's Modified Eagle's Medium (DMEM), other cell lines in this work were grown in RPMI-1640 medium supplemented with 10% fetal bovine serum (FBS) and antibiotics (100 U/mL penicillin, and 100 μg/mL streptomycin). All cell lines were cultured in an incubator with a humidified atmosphere containing 5% CO_2_ at 37 °C.

### Luciferase and MTT assay

Luciferase activity was measured using the Steady-Glo® Luciferase Assay System (Promega, USA). Briefly, PC3-NF-κB-Luc cells were plated in 96-well plates at a density of 5×10^4^ cells/well. After 24 h, the cells were left untreated or treated with TNF-α (20 ng/ml) for 30 min and then lycorine (0 μM, 1 μM, 5 μM, 10 μM, 20 μM) and TPCA-1 (2 μM) were added for further 12 h. At the end of treatment, an equal volume of Steady-Glo Reagent was added into the culture medium in the microplate, and luciferase activity was measured using a Wallac1420 VICTOR microplate reader (Perkin-Elmer, Wellesley, MA, USA).

Cell viability was examined using the MTT method. Briefly, cells were plated in 96-well plates at a density of 5×10^4^ cells/well for 24 h and then treated with lycorine (0 μM, 1 μM, 5 μM, 10 μM, 20 μM). At the end of each treatment, 20 μL of MTT solutions (5 mg/ml) was added into each well and incubated for 2 h at 37 °C. After the medium was removed, 100 μL DMSO was added to each well to dissolve water-insoluble formazan crystals. The optical density of each well was measured with a Wallac1420 VICTOR microplate reader at 490 nm (Perkin-Elmer, Wellesley, MA, USA).

### Western blotting analysis

Western blotting analysis was performed as described in our previous studies 17, 18. Briefly, cells were washed with pre-cold PBS and lysed in RIPA lysis buffer containing 50 mM Tris-HCl (pH 7.5), 150 mM NaCl, 1% NP-40, 0.5% DOC, 0.1% SDS, 5 mM EDTA, 1 mM EGTA, 20 mM NaF, 2 mM sodium orthovanadate and Protease Inhibitor Cocktail (Roche). Total protein concentration was determined using a BCA protein kit (Thermo Fisher Scientific, MA, USA) and 30 μg total protein samples were run on SDS-PAGE and then transferred to PVDF membranes. After blocking with 5% non-fat milk in TBST (0.1%), the membranes were probed with specific primary and secondary antibodies. The membranes were washed five times in TBST and then incubated with an enhanced chemiluminescence western detection system (Perkin-Elmer Life Sciences, Boston, MA, USA) and exposed to X-ray film.

### Immunofluorescence analysis

Cells were plated onto gelatin coated cover slips (1×10^5^/ slips) and the cells were or were not treated Lycorine. After 6 h, cells were stimulated with TNF-α 30 min. After treatments, the cells were fixed with 4% paraformaldehyde for 10 min at room temperature, and then incubated in blocking buffer for 1 h at 37 °C. After removing the blocking buffer, the cells were incubated with primary antibody overnight at 4 °C. After washing them three times with cold PBS, the Cy3-conjugated secondary antibody was added and incubated for 1 h at 37 °C. DAPI was used for nucleus staining. Photographs were obtained with an Olympus BX53 microscope.

### Trans well migration analysis

The *in vitro* migration assay was performed using Trans-well Boyden chamber. Briefly, 1×10^6^ cells were seeded per well in the up chamber with Lycorine or DMSO in serum-free medium. Serum-containing medium was placed in the lower chamber to act as a chemotactic attractant. After treating with Lycorine 12 h or 24 h, the cells were washed with PBS twice and then fixed with paraformaldehyde for 10 min. Cells were then stained with 1% crystal violet for 10 min. After washing with PBS, cells were photographed under the microscope (10 × objectives). To determine how many cells migrated, 100 μL of 33% acetic acid solution was added into the wells and optical density was measured with a Wallac1420 VICTOR microplate reader at 570 nm.

### Flow cytometer analysis

Presidium iodide (PI) staining cell cycle analysis was performed using a flow cytometer. Briefly**,** cells were treated with Lycorine as indicated, and then 10^6^ cells were collected in centrifuge tubes. After being fixed with 75% alcohol overnight at 4 °C, the cells were washed with cold PBS three times. Supernatant was removed and presidium iodide (50 ng/mL of RNase A and 50 μg/mL of PI) working solution was added for staining 30 min at 4 °C. The distribution of the cell cycle was measured by a flow cytometer (LSRFortessa, BD biosciences).

Flow cytometric analysis of cell apoptosis was performed by using the annexin V and PI staining according to the manufacturers' guidelines. The cells were treated with or without Lycorine for 48 h, and then collected. After washing with cold PBS twice, Annexin-FITC was added into the cells. After 15 min, the cells were incubated with PI for 5 min at 4 °C in dark. Cell apoptosis was evaluated by using a Flow cytometer (LSRFortessa, BD biosciences).

### Xenograft studies

The *in vivo* antitumor activity of Lycorine was evaluated in a xenograft model of human prostate cancer. C57/BL male mice were supplied by the experimental animal center of Lanzhou University and the care and use of mice complied with the guidelines for the Care and Use of Laboratory Animals. Briefly, 10^6^ of RM-1 cells were injected into the armpits of mice right legs. After the tumor volumes reached 20 mm^3^, 15 of the tumor bearing mice were divided into three groups: 0.4% DMSO, 5 mg/kg/day Lycorine, and 10 mg/kg/day Lycorine and the mice were treated daily by gavage for two weeks. The mice were weighted every two days. At the end of the treatment, the mice were sacrificed and the tumor tissue was excised for tumor growth analysis (tumor weight) H&E staining, Immunohistochemistry and Immunofluorescence analysis.

### Statistical analysis

All experiments were performed at least three times and all the quantitative data are presented as means ±S.D. The statistical analysis was performed using the SPSS 19.0 software. *p* < 0.05 was considered as significant and *p* < 0.01 as highly significant.

## Results

### Lycorine inhibits NF-κB-regulated luciferase reporter gene activity

**Figure 1:**
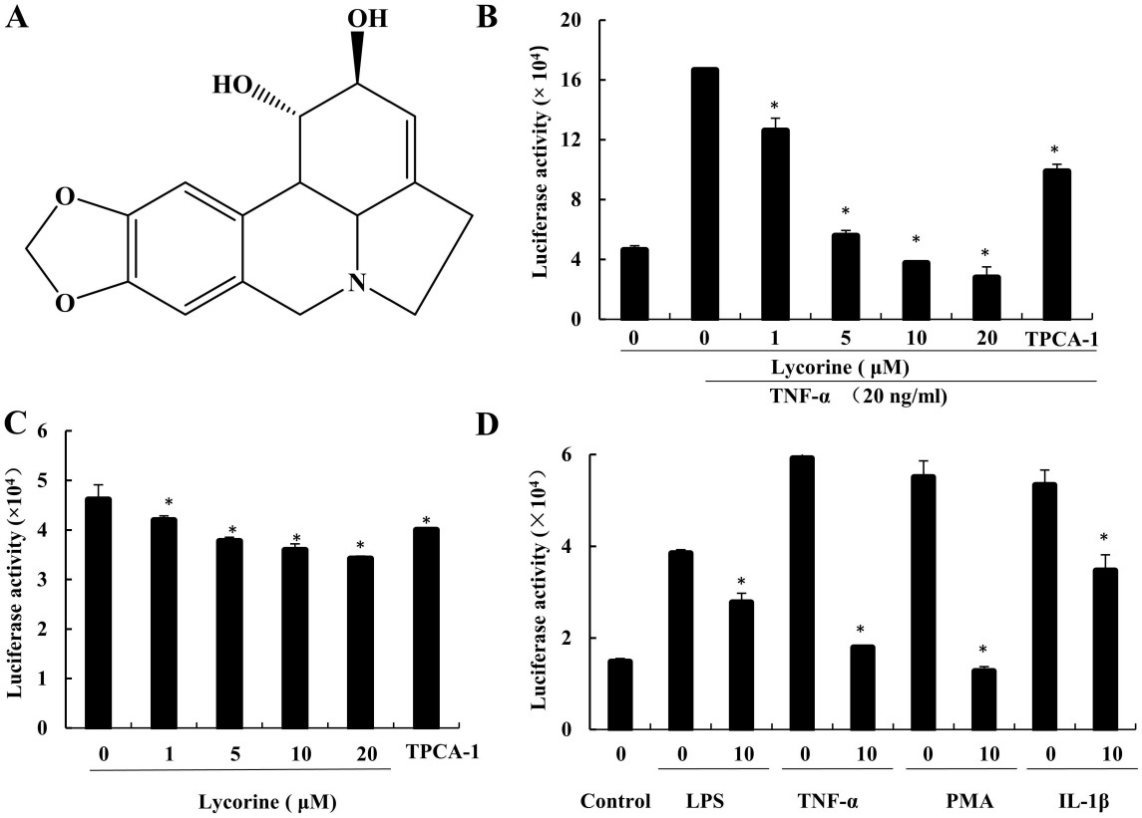
** Lycorine inhibited NF-κB activity. (A)** Structure of lycorine. **(B)** Lycorine inhibited TNF-α induced NF-κB activity. The PC3-NF-κB-Luc were left untreated or exposed to TNF-α (20 ng/ml) for 12 h. Then, PC3-NF-κB-Luc cells were treated with Lycorine at final concentration as indicated. TPCA-1 (2 µM) is a specific inhibitor of NF-κB for positive control and DMSO as vehicle. Luciferase activity was measured using Steady-Glo® Luciferase Assay System (Promega). The values are mean±S.D. for three independent replicates. **(C)** Lycorine inhibited constitutive NF-κB-dependent luciferase activity. PC3-NF-κB-Luc cells were treated as describe above with lycorine in different concentration. **(D)** Lycorine inhibited NF-κB signaling activity via different treatments stimulating. Lycorine concentration is 1 µM. PC3 cells were treated with LPS (1 µg/ml), TNF-α (20 ng/ml), PMA (30 ng/ml) and IL-1β (20 ng/ml) for inducing NF-κB signaling pathway activity. *P<0.05, **P<0.001.

In order to investigate the anticancer effect of Lycorine in prostate cancer, we determined its ability to inhibit NF-κB signaling activity using prostate cancer PC3 cells that stably express the NF-κB regulated luciferase reporter gene (PC3-NF-κB-Luc) 12. With this reporter gene assay, we found that Lycorine (1-20 μM) inhibited TNF-α induced NF-κB signaling activity as well as constitutive NF-κB signaling activity in PC3-κB-Luc cells in a dose dependent manner (Figure 1A, B and C).

In addition, since IL-1β, PMA and LPS can also activate the canonical NF-κB signaling through different mechanisms 3, 21-23, we next determined the ability of Lycorine to inhibit the activation of NF-κB signaling induced by these reagents in PC3-κB-Luc cells. As shown in Figure 1D, similar to TNF-α treatment, the NF-κB signaling was activated by treating PC3-κB-Luc cells with IL-1β, PMA and LPS, but this activation of NF-κB signaling was suppressed when the cells were incubated with Lycorine (1 μM). These results suggest that Lycorine can inhibit the constitutive NF-κB activation as well as the induced NF-κB activation in prostate cancer cells.

### Lycorine inhibits TNF-α induced NF-κB signaling transduction

**Figure 2:**
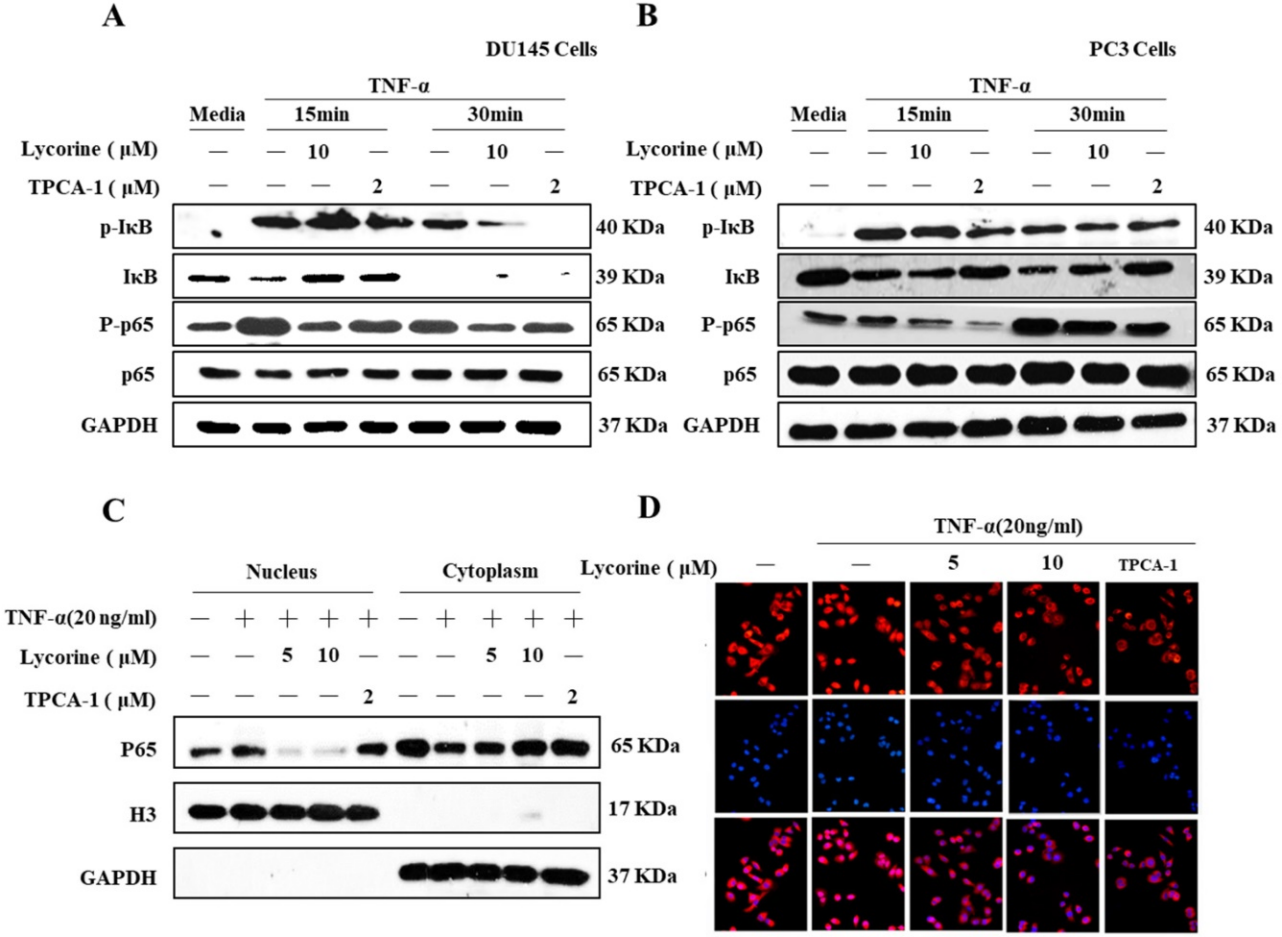
** Lycorine inhibited phosphorylation p65 and IκB degradation. (A, B)** Lycorine inhibited p65 and IκB phosphorylation. Prostate cells DU145 and PC3 cells were treated or untreated with TNF-α (20 ng/ml) for 30 min. After that, the cells were treated or untreated with Lycorine at final concentration as indicated for 12 h. Then cells were left untreated or exposed to Whole-cell extracts were prepared and analyzed by Western blot analysis using the indicated antibodies. **(C)** Lycorine inhibited p65 nuclear translocation in PC3 cells. PC3 cells were treated or untreated with TNF-α for 30 min, and then exposed to DMSO, lycorine or TPCA-1. Cytoplasmic and nuclear extracts representing equal numbers of cells were analyzed by Western blot with the indicated antibodies. **(D)** Lycorine inhibited p65 expression in PC3 cells via immunofluorescence. After treating with TNF-α for 30 min, PC3 cells were treated with DMSO, lycorine (5 µmol/L, 10 µmol/L) or TPCA-1. Immunostaining was performed with specific mouse anti-p65 antibody followed by Cy3-conjugated mouse anti-rabbit immunoglobulins (red) immediately after the treatment.

To further confirm the inhibition effect of Lycorine on the activation of NF-κB, we determined the effect of Lycorine on TNF-α induced signaling transduction of the NF-κB pathway in prostate cancer cells DU145 and PC3. As shown in Figure 2A and Figure 2B, stimulating cells with TNF-α induced a clearly phosphorylation of IκB, its subsequent degradation and phosphorylation of p65 in both cell lines, but pretreating cells with Lycorine or TPCA-1 significantly inhibited the TNF-α induced phosphorylation and degradation of IκB and phosphorylation of p65.

Because p65 translocates into nuclear to induce gene expression after degradation of IκB, we sought to determine whether Lycorine could also inhibit TNF-α induced nuclear translocation of p65. To do this, we pretreated PC3 cells with DMSO, Lycorine or TPCA-1, stimulated them with TNF-α, and then analyzed the p65 protein distribution in cytoplasmic and nuclear fractions using western blotting analysis. After treating the cells with TNF-α, p65 was dramatically translocated into the nucleus (Figure 2C), but the translocation of p65 was blocked in cells pretreated with Lycorine or TPCA-1. Moreover, we validated these results in PC3 cells using immunofluorescence. Compared to the control, we observed a clear localization of p65 (red) in the nucleus of PC3 cells after TNF-α stimulating alone (Figure 2D). Notably, in untreated cell as wells as Lycorine or TPCA-1 pretreated cells, TNF-α failed to induce the translocation of p65 protein from the cytoplasm into the nucleus. These results further demonstrated that Lycorine inhibited NF-κB signaling transduction.

### Lycorine arrests cell cycle in G2/M phase

**Figure 3:**
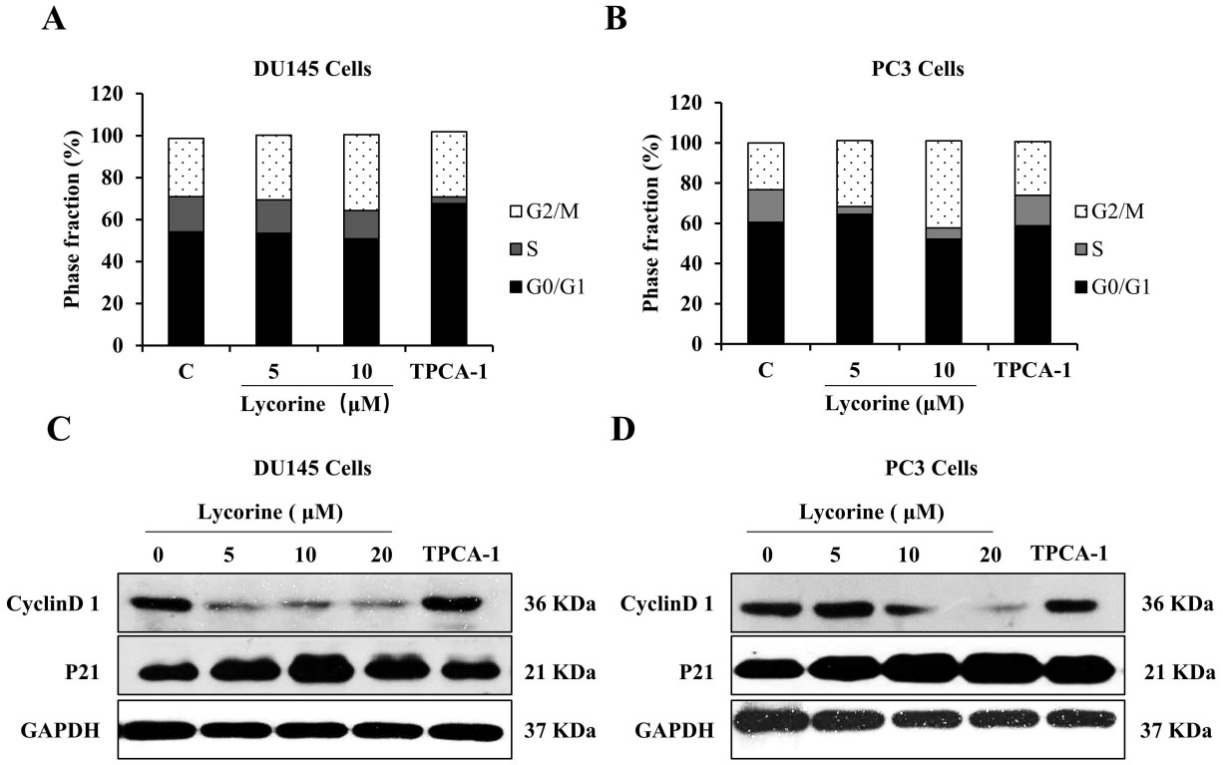
** Lycorine induces G2/M phase cell cycle arrest in DU145 and PC3 cells. (A)** We analyzed cell cycle distribution of DU145 Cells using PI staining. Cells were harvested for cell cycle distribution analysis using presidium iodide staining 24 hours after treatment with lycorine at the concentration as indicated. **(B)** We analyzed cell cycle distribution of PC3 Cells using PI staining. After treatment with lycorine for 24 h, the cells were harvested for cell cycle distribution analysis using presidium iodide staining. **(C)** The expression of cell cycle inhibitory protein p21 and cyclinD1 was detected by western blot in DU145 Cells. **(D)** The expression of cell cycle inhibitory protein p21 and cyclinD1 was detected by western blot in PC3 Cells.

NF-κB regulates the expression of many genes related to cell cycle regulation, which in turn determines cell apoptosis and proliferation. We next performed flow cytometer cycle analysis to determine whether Lycorine could affect the cell cycle progression of PC3 and DU145 cells. Lycorine (5, 10 μM) induced a G2/M phase accumulation of both cell lines in a dose dependent manner, accompanied by a decrease of the proportion of S phase and G0/G1 phase (Figure 3A and B). Furthermore, to validate the cell cycle arresting effect induced by Lycorine, we analyzed the protein expression of cycle related genes cyclinD1 and p21. Western blotting analysis showed that Lycorine dramatically decreased the protein expression of cyclinD1 in a dose dependent manner, while it significantly increased the protein expression of p21 in both PC3 and DU145 cell lines (Figure 3C and D). These results clearly suggest that Lycorine is able to arrest the cell cycle at G2/M phase.

### Lycorine induces cell apoptosis and inhibits cell migration

**Figure 4:**
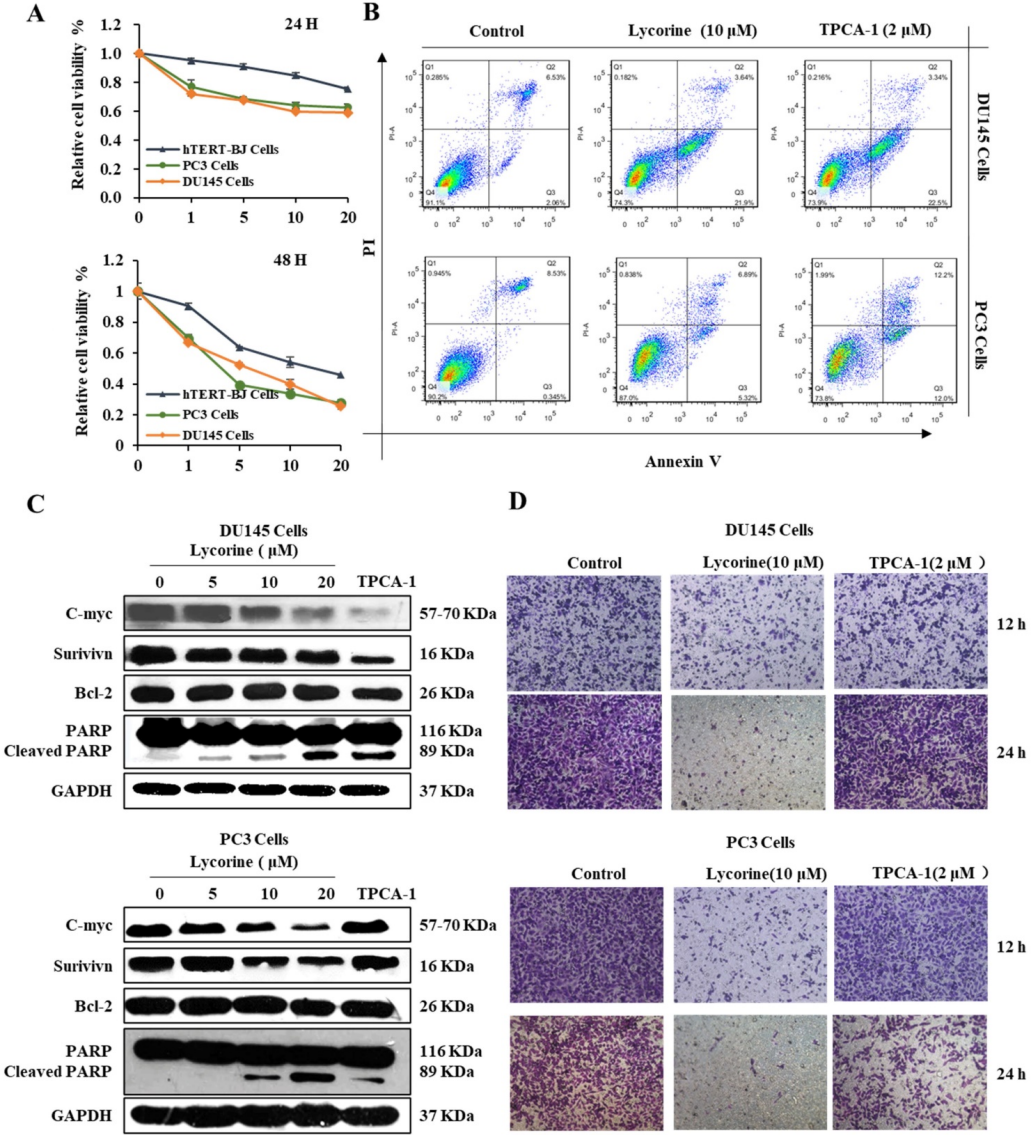
** Lycorine induced prostate cancer cell apoptosis. (A)** DU145 cells and PC3 cells were treated with Lycorine at final concentrations as indicated. Cell viability was determined using MTT Assay After treated with lycorine for 24 hours. **(B)** Cells were harvest for apoptotic analysis using Annexin V-FITC staining. **(C)** Cells were harvest after treat with lycorine for 24 hours, and then analyzed by western blot, GAPDH was used as control. **(D)** Lycorine inhibited DU145 and PC3 cells migration. (A) PC3 and DU145 cells were plated in the upper chamber without FBS, and tested with or without lycorine. And 1640 with FBS were plated in the lower chamber. On the time, after removing the supernatant, cells were stained with crystal. And then record the data with a microscope.

Given that Lycorine is able to arrest the cell cycle, we determined whether inhibiting NF-κB by Lycorine could induce apoptosis in prostate cancer cells. To determine the anticancer effect of Lycorine *in vitro*, we analyzed the cell viability of human prostate cancer cell lines using the MTT assay, which revealed that Lycorine has a more potent inhibitory effect on viability of DU145 and PC3 cells than hTERT-BJ cells (Figure 4A). To confirm that this cell viability inhibition by Lycorine is because of cell apoptosis, we further performed the annexin-V-FITC and Propidium iodide (PI) staining analysis of PC3 and DU145 cells. We found that treating cells with Lycorine resulted in an increase of the percentage of early apoptotic DU145 cells and PC3 cells to 21% and 5% respectively, compared with control cells (Figure 4B). Further, we determined the effect of Lycorine on anti-apoptotic proteins c-myc, survivin and Bcl-2. Western blotting analysis indicated that Lycorine showed a potent decrease effect on the expression of c-myc, but it has slightly detectable influence on the expression of survivin and Bcl-2 in both Du145 and PC3 cell lines. We also studied the ability of Lycorine to induce the cleavage of poly (ADP-ribose) polymerase (PARP), which is commonly used as a marker for cells undergoing apoptosis. Notably, cleavage of PARP was significantly induced by Lycorine in both DU145 and PC3 cells (Figure 4C).

In addition, we analyzed the ability of Lycorine to inhibit the migration ability of PC3 and DU145 cells using Boyden chamber trans-well assay. As shown in Figure 4D, we observed less percentage of DU145 cells as well as PC3 cells in the lower chamber, indicating that Lycorine inhibited cell migration of DU145 and PC3 cells. These results demonstrated that Lycorine can induce apoptosis of PC3 and DU145 cells and inhibit cell migration of PC3 and DU145 cells.

### Lycorine suppresses tumor growth in RM-1 subcutaneous cancer xenograft mouse model

**Figure 5:**
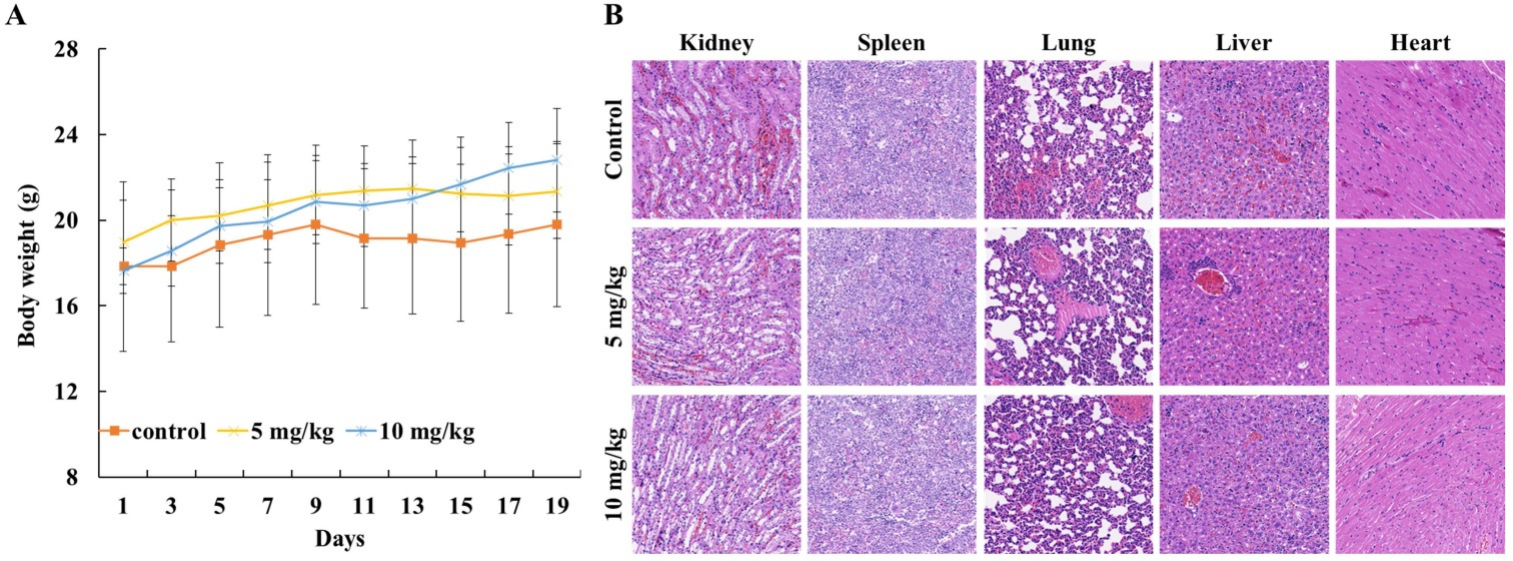
**The biological toxicity of lycorine* in vivo*. (A)** Mouse weight. The mouse were weighted every two days after injected with RM-1. **(B)** HE-staining (×20) showed the major organs (kidney, spleen, lung, liver and heart) of mouse.

**Figure 6:**
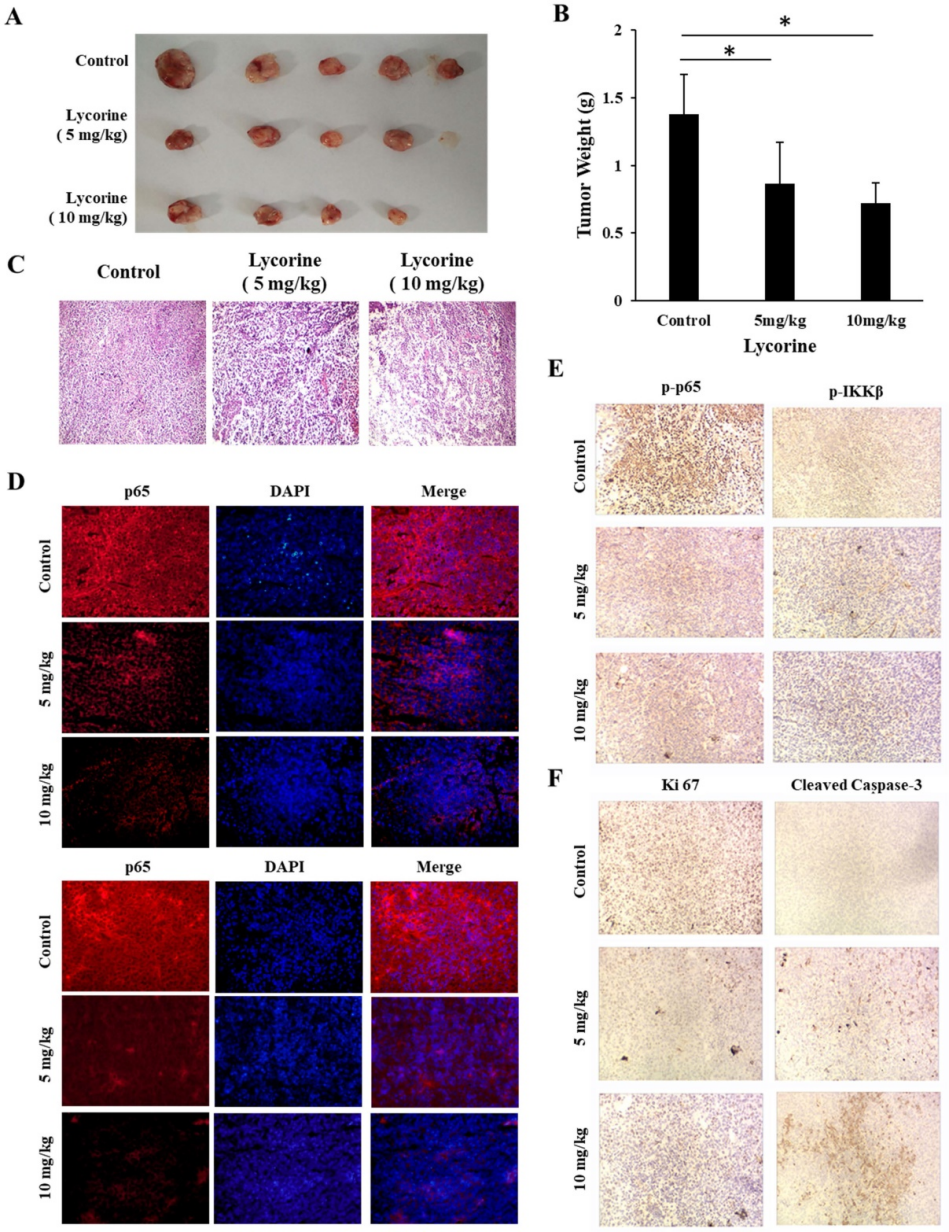
** Lycorine inhibited tumor growth *in vivo*. (A)** Images of the tumor. C57/BL male mouse were injected with RM-1 to establish tumor xenograft mode. Then lycorine was given every day. When the tumor-burdened group's tumor mass reached 1 cm^3^, mice were sacrificed, and the tumor was taken out and weighted. **(B)** Tumor weight. **(C)** HE-staining (×20) showed the tumor tissues of mice. **(D)** Immunofluorescence assay analysis were used to detect the expression of p65 (×20). **(E)** Immunohistochemistry analysis were used to detect the expression of p-p65 and p-IKKβ (×20). **(F)** Lycorine inhibited apoptosis-related proteins *in vivo* by Immunohistochemistry assay. *P<0.05, **P<0.001.

To evaluate the *in vivo* anticancer activity of Lycorine, we injected C57/BL mice with RM-1 cells to generate a RM-1 subcutaneous cancer xenograft mouse model and then the mice were fed with DMSO or Lycorine. As shown in Figure 5A and B, we found that treatment of Lycorine shown no noticeable toxicity to mouse weight and the major organs. Moreover, lycorine (5 mg/kg, 19 days) led to significantly reduce of tumor weight comparing with mouse treated with DMSO (Figure 6 A and B). H&E staining of tumor tissue showed that the number of tumor cells in the Lycorine treated groups decreased significantly (Figure 6C), compared to control.

We next examined the effects of Lycorine on NF-κB signaling pathway in solid tumor tissue using immunofluorescence and immunohistochemistry analysis. As shown in Figure 6 D and E, it was found that both phosphorylation and total protein level of p65 and IKKβ dramatically decreased in the tumor tissue from Lycorine treated groups as compared with the control group. In addition, we also found increased expression of cleaved caspase-3, and decreased expression of KI-67 in Lycorine treatment group (Figure.6F). Taken together, these results showed that Lycorine significantly suppressed tumor growth and inhibited IKK/NF-κB signal pathway *in vivo*.

## Discussion

NF-κB transcription factors regulate the expression of more than 150 genes, which are involved in the development of various malignant tumors 19. The progression of non-androgen dependent prostate cancer, including tumor growth, anti-apoptosis, metastatic and PSA recurrence, correlates with the abnormal activation of NF-κB 10, 20. Identifying the NF-κB signaling pathway inhibitors could help find potent drugs for prostate cancer treatment. In this study, we employed a NF-κB regulated luciferase reporter to identify anti-cancer NF-κB inhibitors in prostate cancer cells and found that Lycorine is a very potent inhibitor of NF-κB in prostate cancer.

A variety of agents can activate the NF-κB signaling through different mechanisms. Some of these agents, including TNF-α, LPS, PMA and IL-1β, can induce NF-κB activation through the IKK kinase complex. In the present work, we showed that Lycorine significantly inhibited NF-κB regulated luciferase activities which were induced by TNF-α, LPS, PMA and IL-1β. Moreover, western blotting and immunochemistry analysis of NF-κB signaling transduction showed that Lycorine suppressed IκB-α phosphorylation, IκB-α degradation, phosphorylation and nuclear translocation of p65 in prostate cancer cells. In addition, we also found that different sensitivity of different cell lines to lycorine, which has been consisted with previous study 16. These collected findings suggested that Lycorine may inhibit NF-κB signaling pathway by targeting the IKK kinase complex.

Numerous findings have demonstrated that blocking the NF-κB signaling pathway can efficiently suppress cancer progress by inhibiting cell growth, arresting cell cycle and promoting apoptosis 21, 22. In this study, we found that Lycorine induced cell cycle arrest and promoted cell apoptosis in PC3 and DU145 cells as demonstrated in flow cytometric analysis. Because overexpression of p21 can lead to cell cycle arrest in G2/M phase by down-regulating CDK activity 23, 24, we tested the effect of Lycorine on p21 expression. After treating cells with Lycorine, the expression of p21 was up-regulated, while the expression of CyclinD1 was significantly down-regulated. In addition, Lycorine can promote PARP cleavage and suppress the expression of anti-apoptosis protein c-myc, Bcl2 and survivin, indicating apoptosis occurred in these cells.

Evidence has suggested that Lycorine showed anti-tumor activity with a low cytotoxicity *in vivo* 14, 15, 25, and mice treated with Lycorine (15 mg/kg for 24 days) did not show an obvious toxic effect 14. In the present work, we showed that Lycorine significantly suppressed tumor growth in a C57/BL-RM-1 mice model without obviously effecting mice weight. Consistent with *in vitro* anti-tumor analysis, Lycorine decreased the phosphorylation of p65 and IKKβ but increased the cleavage of caspase3 and KI67. These data indicated that Lycorine suppressed tumor growth by down-regulating the NF-κB signaling activity.

In summary, this study demonstrated that Lycorine can inhibit the NF-κB signal pathway in prostate cancer cells and showed promising antitumor activity *in vitro* and *in vivo*. These results suggest that Lycorine has the potential to be developed into effective and safe anticancer drugs.

## Abbreviations

NF-κB: nuclear factor kappa-B
TNF-α: tumor necrosis factor-α
LPS: Lipopolysaccharide
PMA: phorbol myristate acetate
IL-1β: interleukin-1β
PARP: poly ADP-ribose polymerase
STAT: signal transducer and activator of transcription
